# Long-term oncological outcomes of endoscopic full-thickness resection after previous incomplete resection of low-risk T1 CRC (LOCAL-study): study protocol of a national prospective cohort study

**DOI:** 10.1186/s12876-022-02591-5

**Published:** 2022-12-13

**Authors:** L. W. Zwager, L. M. G. Moons, A. Farina Sarasqueta, M. M. Laclé, S. C. Albers, R. Hompes, K. C. M. J. Peeters, F. C. Bekkering, J. J. Boonstra, F. ter Borg, P. R. Bos, G. J. Bulte, E. A. R. Gielisse, W. L. Hazen, W. R. ten Hove, M. H. M. G. Houben, M. W. Mundt, W. B. Nagengast, L. E. Perk, R. Quispel, S. T. Rietdijk, F. J. Rando Munoz, R. J. J. de Ridder, M. P. Schwartz, R. M. Schreuder, T. C. J. Seerden, H. van der Sluis, B. W. van der Spek, J. W. A. Straathof, J. S. Terhaar Sive Droste, M. S. Vlug, W. van de Vrie, B. L. A. M. Weusten, T. D. de Wijkerslooth, H. J. Wolters, P. Fockens, E. Dekker, B. A. J. Bastiaansen, the Dutch eFTR Working Group

**Affiliations:** 1 Department of Gastroenterology and Hepatology, Amsterdam University Medical Centers Location University of Amsterdam, Meibergdreef 9, Amsterdam, The Netherlands; 2 Amsterdam Gastroenterology Endocrinology Metabolism, Amsterdam, The Netherlands; 3 Cancer Center Amsterdam, Amsterdam, The Netherlands; 4 Department of Gastroenterology and Hepatology, University Medical Center Utrecht, Utrecht, The Netherlands; 5 Department of Pathology, Amsterdam University Medical Center, Amsterdam, The Netherlands; 6 Department of Pathology, University Medical Center Utrecht, Utrecht, The Netherlands; 7 Department of Surgery, Amsterdam University Medical Center, Amsterdam, The Netherlands; 8 Department of Surgery, Leiden University Medical Center, Leiden, The Netherlands; 9 Department of Gastroenterology and Hepatology, IJsselland Hospital, Capelle Aan Den Ijssel, The Netherlands; 10 Department of Gastroenterology and Hepatology, Leiden University Medical Center, Leiden, The Netherlands; 11 Department of Gastroenterology and Hepatology, Deventer Hospital, Deventer, The Netherlands; 12 Department of Gastroenterology and Hepatology, Gelderse Vallei, Ede, The Netherlands; 13 Department of Gastroenterology and Hepatology, Radboud University Medical Center, Nijmegen, The Netherlands; 14 Department of Gastroenterology and Hepatology, Rode Kruis Hospital, Beverwijk, The Netherlands; 15 Department of Gastroenterology and Hepatology, Elisabeth Tweesteden Hospital, Tilburg, The Netherlands; 16 Department of Gastroenterology and Hepatology, Alrijne Medical Group, Leiden, The Netherlands; 17 Department of Gastroenterology and Hepatology, Haga Teaching Hospital, the Hague, The Netherlands; 18 Department of Gastroenterology and Hepatology, Flevoziekenhuis, Almere, The Netherlands; 19 Department of Gastroenterology and Hepatology, University Medical Center Groningen, Groningen, The Netherlands; 20 Department of Gastroenterology and Hepatology, Haaglanden Medical Center, The Hague, The Netherlands; 21 Department of Gastroenterology and Hepatology, Reinier de Graaf, Delft, The Netherlands; 22 Department of Gastroenterology and Hepatology, OLVG, Amsterdam, The Netherlands; 23 Department of Gastroenterology and Hepatology, Nij Smellinghe Hospital, Drachten, The Netherlands; 24 Department of Gastroenterology and Hepatology, Maastricht University Medical Center, Maastricht, The Netherlands; 25 Department of Gastroenterology and Hepatology, Meander Medical Center, Amersfoort, The Netherlands; 26 Department of Gastroenterology and Hepatology, Catharina Hospital, Eindhoven, The Netherlands; 27 Department of Gastroenterology and Hepatology, Amphia Hospital, Breda, The Netherlands; 28 Department of Gastroenterology and Hepatology, Isala Clinics, Zwolle, The Netherlands; 29 Department of Gastroenterology and Hepatology, Noordwest Hospital Group, Alkmaar, The Netherlands; 30 Department of Gastroenterology and Hepatology, Màxima Medical Center, Veldhoven, The Netherlands; 31 Department of Gastroenterology and Hepatology, Jeroen Bosch Hospital, S’ Hertogenbosch, The Netherlands; 32 Department of Gastroenterology and Hepatology, Dijklander Hospital, Hoorn, The Netherlands; 33 Department of Gastroenterology and Hepatology, Albert Schweitzer Hospital, Dordrecht, The Netherlands; 34 Department of Gastroenterology and Hepatology, St. Antonius Hospital, Nieuwegein, The Netherlands; 35 Department of Gastrointestinal Oncology, Netherlands Cancer Institute/Antoni Van Leeuwenhoek, Amsterdam, The Netherlands; 36 Department of Gastroenterology and Hepatology, Martini Hospital, Groningen, The Netherlands

**Keywords:** Endoscopic full-thickness resection, T1 colorectal cancer, Colorectal cancer, Minimal invasive local treatment options

## Abstract

**Background:**

T1 colorectal cancer (CRC) without histological high-risk factors for lymph node metastasis (LNM) can potentially be cured by endoscopic resection, which is associated with significantly lower morbidity, mortality and costs compared to radical surgery. An important prerequisite for endoscopic resection as definite treatment is the histological confirmation of tumour-free resection margins. Incomplete resection with involved (R1) or indeterminate (Rx) margins is considered a strong risk factor for residual disease and local recurrence. Therefore, international guidelines recommend additional surgery in case of R1/Rx resection, even in absence of high-risk factors for LNM. Endoscopic full-thickness resection (eFTR) is a relatively new technique that allows transmural resection of colorectal lesions. Local scar excision after prior R1/Rx resection of low-risk T1 CRC could offer an attractive minimal invasive strategy to achieve confirmation about radicality of the previous resection or a second attempt for radical resection of residual luminal cancer. However, oncologic safety has not been established and long-term data are lacking. Besides, surveillance varies widely and requires standardization.

**Methods/design:**

In this nationwide, multicenter, prospective cohort study we aim to assess feasibility and oncological safety of completion eFTR following incomplete resection of low-risk T1 CRC. The primary endpoint is to assess the 2 and 5 year luminal local tumor recurrence rate. Secondary study endpoints are to assess feasibility, percentage of curative eFTR-resections, presence of scar tissue and/or complete scar excision at histopathology, safety of eFTR compared to surgery, 2 and 5 year nodal and/or distant tumor recurrence rate and 5-year disease-specific and overall-survival rate.

**Discussion:**

Since the implementation of CRC screening programs, the diagnostic rate of T1 CRC is steadily increasing. A significant proportion is not recognized as cancer before endoscopic resection and is therefore resected through conventional techniques primarily reserved for benign polyps. As such, precise histological assessment is often hampered due to cauterization and fragmentation and frequently leads to treatment dilemmas. This first prospective trial will potentially demonstrate the effectiveness and oncological safety of completion eFTR for patients who have undergone a previous incomplete T1 CRC resection. Hereby, substantial surgical overtreatment may be avoided, leading to treatment optimization and organ preservation.

*Trial registration* Nederlands Trial Register, NL 7879, 16 July 2019 (https://trialregister.nl/trial/7879).

## Background

### Background and rationale

The implementation of colorectal cancer (CRC) screening programs worldwide has led to a significant increase in the detection of T1 CRC [1–5]. These early cancers have a limited risk for lymph node metastasis (LNM), varying between 1 and 16% depending on histopathological risk features and thus can potentially be cured by endoscopic resection [6–9]. Endoscopic resection is an attractive treatment option compared to radical surgery because of its low morbidity and mortality rates and substantially lower costs [10–14]. However, endoscopic resection can only be accepted as final treatment when the risk for LNM or local recurrence do not outweigh surgery associated mortality (1.7%) and disease recurrence despite oncologic surgery (2 to 5%) [10–14]. Treatment recommendations either to proceed with additional oncologic surgery or surveillance alone after endoscopic resection, mainly depend on the presence of histopathological risk factors for local recurrence and LNM [15]. Previous studies have shown that the risk of local recurrence and LNM is very low in absence of the following high-risk features: poor differentiation, lymphovascular invasion, deep submucosal invasion (≥ 1000 µm), high-grade tumor budding (grade 2 or 3) and a positive resection margin (R1/Rx resection) [6, 7, 9, 16–18]. If one of these high-risk features is present, patients are currently counselled for additional oncological surgery according to current guidelines [19, 20].

Positive polypectomy margin status (i.e. positive resection margin < 0.1 mm (R1) or indeterminate resection margin (Rx) is considered a strong risk factor for residual disease in the colon wall and local recurrence [6, 21–26]. Current guidelines therefore advise additional oncological surgery, even in the absence of unfavourable histological factors [18, 27]. However, even in the presence of high-risk factors for LNM, residual disease is noted in less than 20% of patients undergoing additional surgery. Therefore, a surgical resection can be considered potential overtreatment, especially in patients without increased risk for LNM [6, 21, 22, 24]. Despite this apparent surgical overtreatment, a certain number of patients are at risk of having residual cancer with risk of cancer dissemination, when additional oncologic surgery is not performed. Among these patients, CRC-related mortality was reported to be as high as 42% [15]. However, the potential impact of implementation of strict surveillance protocols on earlier diagnosis of cancer recurrence to allow for curative salvage therapy is currently unknown.

In daily practice, real-time endoscopic recognition of T1 CRC is challenging. Recent Dutch studies demonstrated that endoscopists recognized only 22–39% of all diagnosed T1 CRC as being cancer before endoscopic resection [28, 29]. As result of suboptimal diagnosis, 41% missed potential endoscopic cure due to the selection of inappropriate endoscopic techniques [15, 30]. Conventional polypectomy techniques like piecemeal or *en bloc* snare coagulation often hamper adequate histological assessment leading to uncertainty about the completeness of excision or the presence of histological risk factors. To achieve definite histopathological confirmation about radicality of the resection, or a second chance for radical resection in case of remaining cancer within the bowel wall, an additional endoscopic *en bloc* resection of the previous resection scar might be an attractive strategy. Until recently, no safe endoscopic *en bloc* re-resection technique was available for the re-resection of scar tissue or non-lifting, incomplete resected lesions. The introduction of endoscopic full-thickness resection (eFTR) has expanded the therapeutic endoscopic armamentarium and several large-scale clinical studies have now established its feasibility and safety for complex colorectal lesions [31–34]. The eFTR-technique allows a transmural local excision of the scar and therefore can potentially serve as a valid option for curative completion treatment following incomplete T1 CRC resection, potentially avoiding unnecessary surgical risks [35–40].

eFTR is currently increasingly used for this indication in daily practice. Our previous multicenter feasibility study showed that eFTR of scars of previous R1/Rx resected T1 CRC was technically feasible in 85% with promising short-term results. [25] However, long-term oncologic safety has not been established. By implementing completion eFTR in a prospective national cohort study followed by protocolized surveillance, we aim to assess its feasibility and long-term oncologic safety after previous incomplete resection of low-risk T1 CRC.

### Objectives

This project aims to investigate the feasibility and oncological safety of endoscopic full-thickness resection (eFTR) as minimal invasive completion treatment after previous R1/Rx endoscopic resection of low-risk T1 CRC. We hypothesize that completion eFTR is feasible in ≥ 80% of patients with a low risk of luminal cancer recurrence < 2%.

### Trial design

This nationwide prospective observational multicenter study aims to investigate the feasibility and oncological safety of eFTR as completion treatment after previous potential incomplete resection of low-risk T1 CRC. Patients referred for completion eFTR for low-risk T1 CRC with positive resection margins < 0.1 mm (R1) or indeterminate margins (Rx) are eligible for participation in this prospective cohort study. Patients will be followed by protocolized surveillance during 5 years.

## Methods

### Study setting

Prospective observational multicenter study in academic and non-academic hospitals throughout the Netherlands.

### Eligibility criteria

#### Inclusion criteria

Patients meeting all of the following criteria will be invited for participation in the study:

- Recent polypectomy/(p)EMR/ESD of T1 CRC *without* the following histological high-risk features*:
  o Poor differentiation
  p Lymphovascular invasion
  q Tumor budding grade 2/3
- This recent polypectomy/(p)EMR/ESD for T1 CRC resulted in positive resection margins < 0.1 mm (R1) or indeterminate resection margins (Rx)
- The resection scar after polypectomy/EMR/ESD is clearly recognized at endoscopy, either by a tattoo or by detecting a scar in the colorectal segment where no other polypectomies were performed
- Diameter of the original lesion ≤ 30 mm
- Diameter of the scar and/or residual lesion ≤ 15 mm
- Interval between index polypectomy/EMR/ESD and additional eFTR at most 12 weeks
- Staging computed tomography (CT) of thorax and abdomen without local lymph node or distant metastases. In case of rectal location an additional magnetic resonance imaging (MRI) of the pelvis is performed at which no local suspicious lymph node(s) detected. If the target lesion is visible on MRI, rectal location is defined as location distal from the sigmoid take off. If not visible on MRI, rectal location is defined as < 15 cm from anal verge on endoscopy.
- Written informed consent provided

*Before inclusion in this study, eligible histology needs to be centrally reviewed by one of the two expert gastrointestinal pathologist. In case of any doubt on the presence of high-risk features, both expert pathologist will have a case discussion in order to reach consensus.

#### Exclusion criteria

Patients meeting any of the following criteria will be excluded from participation in this study:

- If lymphovascular invasion and/or tumor budding grade 2/3 cannot be assessed after prior polypectomy/(p)EMR, patients are *NOT* eligible for inclusion
- The patient is known with at least one of the following conditions:
  o Active inflammatory bowel disease (IBD) in the colorectum
  p Synchronous advanced CRC (defined as CRC in the 5 years before detection of T1 CRC, or elsewhere in the colorectum at the time of detection of T1 CRC)
- Index lesion located < 5 cm of the anal verge or with involvement of the valvula Bauhini or appendiceal orifice
- Age < 18 years
- Pregnancy

### Who will take informed consent?

Local gastroenterologists, gastroenterology fellows, research nurses or other members of the study team in the participating centers will inform eligible patients about the study aims, the prospective data collection, the eFTR procedure and follow up procedures. All patients will receive an informed consent form. Patients will be given as much time as needed to make an informed decision regarding participation.

### Outcomes

The primary outcome is to assess the 2- and 5- year local luminal tumor recurrence rate after scar resection by eFTR following a previous R1/Rx resection of low-risk T1 CRC.

The secondary outcomes are:

1. to assess the feasibility of completion eFTR, defined as a macroscopic complete en bloc scar excision in > 80% of cases
2. to assess the percentage of curative eFTR resections, defined as no residual cancer in the scar or in case any residual cancer a R0 resection for T1 CRC without high-risk features (poor differentiation, lymphovascular invasion and/or high-grade tumor budding (grade 2 or 3)
3. to assess the presence of scar tissue and/or complete scar excision at histopathology
4. to assess the procedure-related adverse event rate and safety of eFTR compared to oncologic surgery in a historical patient-cohort for T1 CRC
5. to assess the 2- and 5- year locoregional nodal and/or distant tumor recurrence rate
6. to assess the 5-year disease-specific survival rate and overall survival rate
7. to assess the 2- and 5-year luminal, nodal and distant tumor recurrence rate in patients not meeting our inclusion criteria, but who did undergo eFTR completion treatment followed by strict surveillance and are included in our observational arm

## Participant timeline

### Treatment of subjects

#### Baseline examinations

At baseline, patients have undergone a polypectomy/EMR/ESD procedure for a T1 CRC with positive resection margins < 0.1 mm (R1) or indeterminate resection margins (Rx).

All original specimens will be centrally reviewed by one of two expert gastrointestinal pathologists at UMC Utrecht or Amsterdam UMC. Additional immunohistochemical staining will only be applied by our expert pathologists when there is suspicion of LVI on the H&E slides. The expert pathologist will review the slide within 5 working days. Afterwards, all slides will return to the original laboratories. In case of doubt on the presence of one or more histological high-risk features, the other expert pathologist will be consulted in order to reach consensus. In case no consensus can be reached, a third expert pathologist will be consulted. If the quality of the specimen does not allow reasonable determination of risk factors, they will be excluded from study participation.

Furthermore, a staging CT thorax-abdomen or X-thorax and CT abdomen is performed. In case of rectal location an additional magnetic resonance imaging (MRI) of the pelvis is performed. If the target lesion is visible on MRI, rectal location is defined as location distal from the sigmoid take off. If not visible on MRI, rectal location is defined as < 15 cm from anal verge on endoscopy. In addition, Carcinoembryonic Antigen (CEA) will be measured.

#### eFTR procedure

All participating endoscopists are trained in ex vivo porcine models and certified to perform eFTR procedures in the Netherlands. All endoscopists have extensive colonoscopy (≥ 1000 procedures) experience. To minimize the risk that outcomes of this study are influenced by a potential learning curve of the performing endoscopist, only endoscopists with sufficient experience (at least 10 personal eFTR procedures) can participate in this study.

Patients will receive standard bowel preparation, which includes oral intake of a split-dose PEG-solution and bisacodyl in accordance with the standard bowel preparation protocol of the participating center. Procedures will be performed in an out-patient setting under deep sedation with propofol or under conscious sedation by a combination of intravenous midazolam and fentanyl. Monotherapy with antiplatelet agents is continued, all other anticoagulants (double antiplatelet therapy or heparin, coumarins, warfarin, NOACs) are temporarily discontinued according to Dutch guideline. Prescription of procedural prophylactic antibiotic therapy is not advised, but is left at discretion of the performing endoscopist [25].

**Fig. 1:**
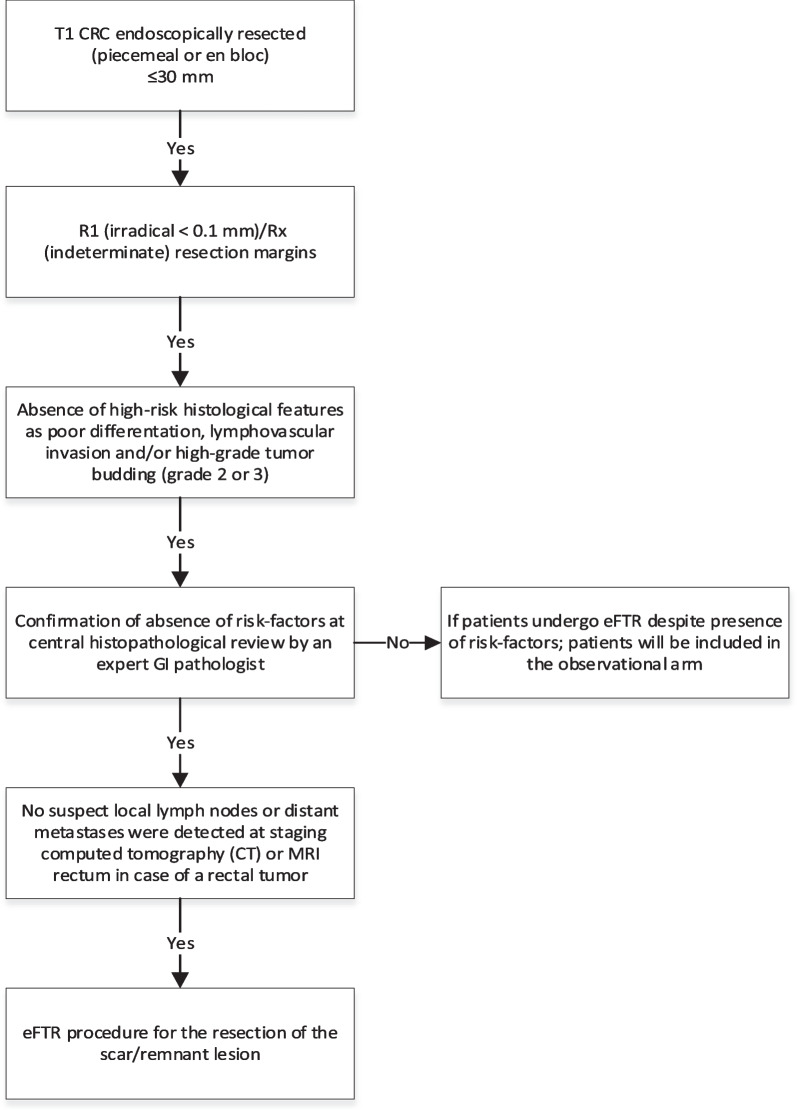
Trial design

**Fig. 2:**
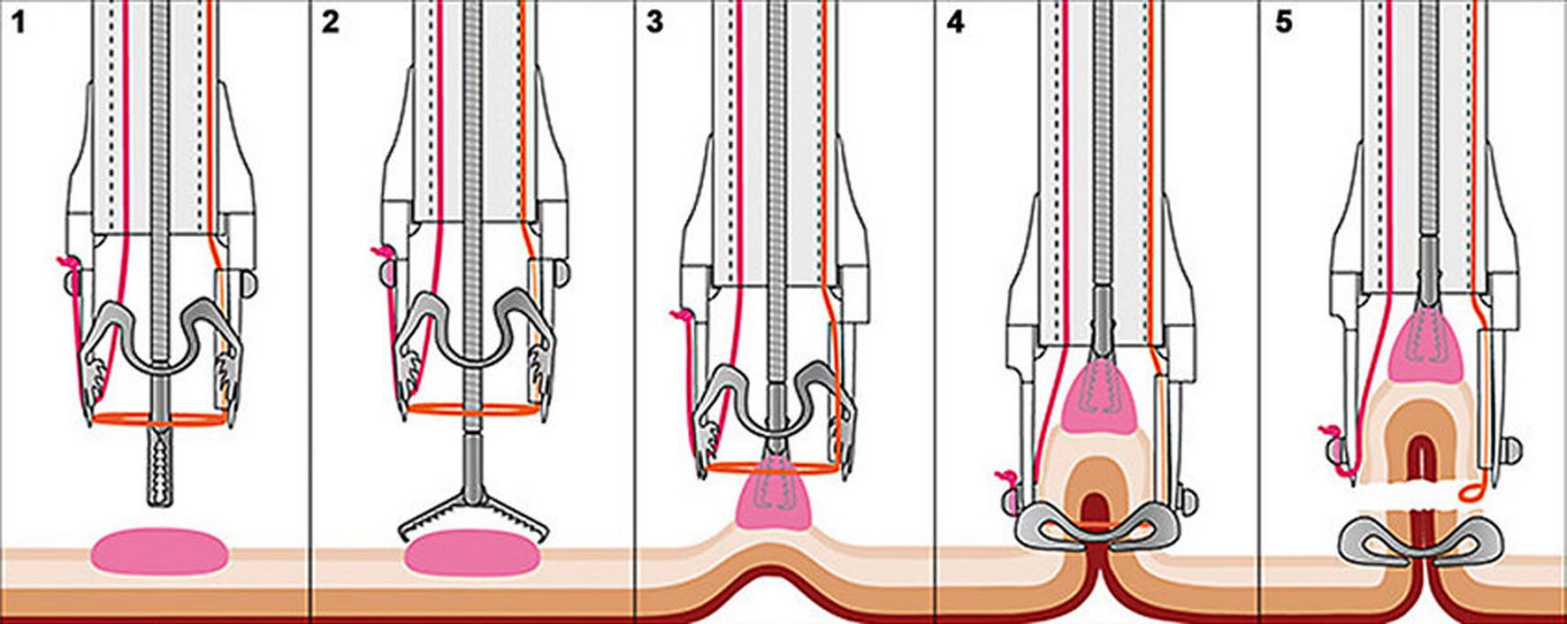
Schematic illustration of the resection procedure (image used with permission from Ovesco Endoscopy AG from www.ovesco.com)

Before the start of the eFTR procedure the scar will be identified using both HD white light endoscopy (WLE) and narrow band imaging (NBI), blue laser imaging (BLI) or other digital chromoendoscopy. Endoscopists preferably take a minimum of three photos of the scar. The lateral margins of the target lesions can be marked by the use of coagulation with the marking probe (Ovesco Endoscopy, Tübingen, Germany) at the discretion of the endoscopist. Hereafter the colonoscope will be extracted from the patient and the FTRD will be mounted. The colonoscope with the mounted FTRD will be reintroduced and advanced to the scar. Then the scar/lesion will be resected as shown in Fig. 1. After collection of the specimen the FTRD will be demounted and the colonoscope will be re-introduced to inspect the resection site. Patients will be discharged according to local protocols. A normal diet will usually be started at postoperative day one (Fig. 2).

#### Histopathology handling

The resection specimen is stretched and pinned down on paraffin or cork before immersed into formalin. Histopathological analysis is performed by a gastrointestinal pathologist according to daily clinical practice. In case of presence of residual adenocarcinoma, deep and lateral resection margins will be assessed as well as all histological risk features (differentiation grade, depth of invasion, lymphovascular invasion and tumor budding (grade 2 or 3)).

In the eFTR specimen, the pathologist will assess the presence of reactive changes compatible with scar tissue in the specimen. Definite histopathological confirmation of complete scar resection is known to be difficult and might not always be possible. In case the pathologist is not able to histologically confirm complete scar resection, further treatment will be based on the macroscopic completeness of scar excision and patients will be followed according to protocol.

In addition, central review of all resected eFTR specimens will be performed after all patients are included in the study. The central review will be performed by two independent expert gastrointestinal pathologists. In this review, the presence of neoplasia, scar tissue and evaluation of completeness of excision regarding both neoplasia and scar will be evaluated by both expert pathologists. In cases without consensus, meetings will be organized to discuss discrepancies to reach consensus. In case no consensus can be reached, a third expert pathologist will be consulted.

H&E slides of all resected eFTR specimens will be collected from the different laboratories and digitalized at the Amsterdam UMC for review. After digitalizing the slides will return to the laboratories to ensure the completeness of pathology archives and optimal patient care.

In case of unexpected findings, the treating physician will be informed to discuss further treatment options with the patients after colorectal MDT discussion. Outcomes of these cases will be recorded in the study database.

### Follow-up

In case the specimen of eFTR contains residual cancer with high-risk features and/or incomplete eFTR resection (Rx/R1), the case should be discussed in a colorectal MDT board for further treatment options depending on histological and endoscopic findings. The same accounts for cases in which completion eFTR of the scar was technically impossible. The outcomes, subsequent treatment and further follow-up procedures of these cases will be recorded in the study database. In this prospective cohort study, the follow-up schedule will be in line with the Dutch Guidelines (in preparation).

For all detected local, nodal and/or distant tumor recurrences during follow-up, the outcomes and subsequent treatment will be recorded in the study database.

#### Endoscopic follow-up

Endoscopic surveillance for lesions located in the colon will be performed by yearly eFTR scar checks at 12, 24, 36, 48 and 60 months. In patients with T1 rectal cancer endoscopic surveillance of the eFTR scar will be performed every 6 months during the first two years followed by yearly scar checks at 36, 48 and 60 months. According to the Dutch guideline, scar checks will be combined with a full colonoscopy at 12 months and 48 months for CRC surveillance [27]. The eFTR scar will be thoroughly inspected with HD WLE and (digital) chromoendoscopy. Endoscopists preferably take a minimum of three photos of the scar. Scar biopsies (3 biopsies) will be taken at every surveillance, even from scars without signs of macroscopic recurrence. Endoscopic and histological findings compatible with granulation tissue or reactive changes can be left untreated. Recurrent/residual low-grade dysplastic adenoma or a sessile serrated lesion can be treated endoscopically with conventional treatment strategies. When high-grade dysplasia or submucosal invasive cancer is found, a tattoo (SPOT) should be placed followed by case discussion in a colorectal MDT.

Local luminal tumor recurrence is defined as the presence of any adenocarcinoma at the site of the eFTR scar at follow-up colonoscopy. Further treatment and follow-up will be planned individually depending on histology and further staging procedures in the participating centers.

#### Radiologic follow-up

For patients who have undergone an eFTR procedure for a tumor located in the colon, a standard protocol CT thorax-abdomen will be performed yearly at 12, 24, 36, 48 and 60 months to evaluate nodal and/or distant tumor recurrence. For rectal location, an MRI of the rectum will be performed every 6 months during the first 2 years after the eFTR procedure followed by yearly MRI scans at 36, 48 and 60 months. Results of our prospective eFTR registry showed the OTSC (clip) in situ after eFTR in only 10.9% after 4 months of follow-up. In the rare cases in which the clip could possibly hamper scar assessment or quality of radiologic follow-up, the OTSC can be removed with a dedicated cutting device. In case of suspected local, nodal and/or distant tumor recurrence the patient will be discussed in a multidisciplinary team meeting to discuss further treatment options according to local clinical practice, which is based on the Dutch CRC guideline [27]. Outcomes and subsequent treatment will be recorded in the study database.

#### Other follow-up

Surveillance with Carcinoembryonic Antigen (CEA) detection in serum will be performed every 6 months for 5 years after the performed eFTR procedure.

#### Observational arm

Patients who will undergo a completion eFTR following a previous incomplete excised T1 CRC, despite being non eligible for study participation (after multidisciplinary team discussion), will be prospectively followed in our study database. Furthermore, treatment outcomes of all non-eligible patients (eg because of the presence of high-risk features or the indetermination of those) will be collected in case no eFTR is performed.

### Sample size

Up-to-date, literature is scarce with regard to local luminal recurrence rates after endoscopic incomplete resection of T1 CRC (R1/Rx resections) for low-risk T1 CRC. A recent study showed local recurrence rates of 4.5% (4/89) after full-thickness resections of incomplete resected presumed low-risk T1 CRC. However, in this study histologic reassessment was not performed before resection and retrospective histologic revision showed high-risk factors for LNM in 3 out of 4 local recurrences [41].

Another study observed a local recurrence rate of 1% (1/102) during almost four years of follow-up after minimally invasive transanal surgery [42]. Considering that literature is limited, the upper limit of its 95% one-sided confidence limit may extend to 4.6%. With already being frequently used in daily practice and considering that the follow-up duration in the current study will by five years (instead of four), we opt to qualify eFTR as unsafe if the upper 95% one-sided confidence limit of its related local recurrence rate after five years extends beyond 6.6%.

A single sample size of 137 patients will have at least 86% power to detect this non-inferiority limit of 6.6% using a one-sided exact test with a significance level of 0.05 for maximum actually observed local recurrence rates of 2% over five years (twice the possibly underestimated local recurrence rate over four years of 1% for minimally invasive surgical treatment). To correct for potential drop-out patients in whom the primary outcome cannot be assessed the sample size will be adjusted to 153 (potential drop-out rate of 10%). These drop-out cases are patients with indication for additional surgery when histopathology review reveals high-risk features (i.e. poor differentiation grade, lymphovascular invasion and/or high-grade tumor budding) for LNM or eFTR is technically impossible. If during 5 years of follow up, more than 5 local luminal recurrences are observed -which implies that the upper limit of the confidence interval will exceed the non-inferiority limit- our study fails to establish oncological safety for completion endoscopic full-thickness resection after previous incomplete resection of low-risk T1 CRC.

### Recruitment

Completion eFTR after incomplete excised T1 CRC is currently offered to patients as an alternative to surgery in clinical practice. After case discussion in a colorectal MDT, recruitment for the study will be performed by gastroenterologists, gastroenterology fellows or research nurses in the participating centers. Eligible patients will be informed about the study aims, the prospective data collection, the eFTR procedure and follow-up procedures and will receive an informed consent form. Patients will hand in the signed informed consent forms to a member of the study team in the participating centers. Patients will be given as much time as needed to make an informed decision regarding participation.

### Plans for assessment and collection of outcomes

Patient data will be collected from the electronic patient databases and recorded in an online secured database (Castor, CIWIT BV, Amsterdam). The PhD student with help of a research nurse will coordinate and regularly visit all participating centers to collect all accomplished data in the database. During surveillance the PhD student will collect all data regarding the surveillance visits in the eCRFs and will monitor study adherence in the participating centers.

### Plans to promote participant retention and complete follow-up

The PhD student and research nurse will monitor study adherence in the participating centers. The PhD student will inform the participating hospitals in case follow-up is not performed on time. Additionally, four patients will be asked to join our study patient advisory board and evaluate yearly patient participation by hearing the report of the research team. The patients’ input helps ensuring optimal patient participation, patient adherence to the five years surveillance and communicate the outcomes to the patient organisation.

### Data management and confidentiality

Patients will be coded by a numeric code (pseudonymised) and only the local investigators and project leader will be the ones to have access to this code. The code lists will be stored digitally on the protected hard disc at the local center that included and treated the subject. The patient data will be recorded in an online secured database (Castor, CIWIT BV, Amsterdam). All study investigators at each study site will have access to the online database of their own site and will be able to insert data.

### Statistical methods for primary and secondary outcomes

For our primary analysis all performed eFTR procedures in patients in whom the primary outcome (luminal local tumor recurrence rate after scar resection) can be assessed will be included. Thus patients in whom the procedure could not be performed for technical reasons or needing additional surgery due to the presence of high-risk features following completion eFTR will not be included for primary analysis.

For secondary analysis regarding feasibility of completion eFTR all included eligible patients will be analysed irrespective of the received completion eFTR or performed additional surgery.

Descriptive statistics will be used to describe patient, colonoscopy and eFTR characteristics for primary and secondary outcomes. Variables will be reported as mean with standard deviation in case of continuous and normally distributed variables, as median with an interquartile range (IQR) in case of non-normally distributed continuous variables, and as percentages in case of count or categorical variables. will use logistic regression with baseline variables to identify predictors for a successful completion eFTR.

The confidence interval of the 5 year disease-specific survival rate and overall survival rate will be calculated. Kaplan–Meier analysis will be used to estimate survival over time.

### Composition of the coordinating centre and trial steering committee

The study team of the coordinating centre consists of the principal investigator, coordinating investigators, PhD student and a research nurse. The PhD student will carry a leading role in coordinating the study and the research nurse will support the PhD student with his or her work. We organize plenary meetings with the eFTR working group twice a year where we will discuss the study progress and results.

### Composition of the data monitoring committee, its role and reporting structure

This study does not involve a data monitoring committee because this study is waived from formal approval by the Ethical Committee of the AMC.

### Adverse event reporting and harms

We will collect all adverse events associated with eFTR for which transfusion, hospitalization (unscheduled post procedural admission) and additional (endoscopic, surgical, radiologic) treatment is needed in our online secured database.

### Frequency and plans for auditing trial conduct

Plenary meetings will be arranged twice a year for the entire duration of the study. We will host each year one online meeting and one “real-life” meeting. Furthermore, we will meet yearly with our patients’ advisory board.

### Plans for communicating important protocol amendments to relevant parties (e.g. trial participants, ethical committees)

If protocol amendments occur during the study, the study team will communicate relevant protocol changes and report substantial changes to the ethics committee.

### Dissemination plans

The progress and results of the study will be discussed in plenary meetings of the eFTR working group twice a year. Due to pleasant and effective collaboration within the eFTR working group, consisting of 29 participating centers the results of this study will be easily adapted in daily clinical practice. On a national basis, the results of this study will be presented during national meetings, which are attended by gastroenterologists, pathologists and surgeons. We expect that implementation of results in the Dutch guideline will be a logical next step. Furthermore, through presentations at national and international meetings and through publications in scientific journals we will raise awareness for the study and its results internationally.

## Discussion

The LOCAL study is the first prospective study that will assess the long-term oncologic safety of completion eFTR as minimally invasive completion treatment for a previous R1/Rx excised low-risk T1 CRC, followed by strict surveillance. In this study we hypothesize that additional scar resection by eFTR is feasible in ≥ 80% of patients with a low risk of luminal cancer recurrence in < 2%. In addition, we hypothesize that active protocolized surveillance will enable curative salvage surgery in case of cancer recurrence.

In the development of the study design, we have chosen for a national prospective cohort study to investigate our hypothesis. Performing a randomized trial on this topic will be difficult for several reasons. First, patients often have a strong preference for a minimal invasive strategy. Besides, not all patients will be good surgical candidates and thus not be eligible for a randomized study. Third, it would take a very long time to complete a randomized study while in the meantime, despite the lack of evidence for its long-term oncologic safety, completion eFTR will already be offered to patients in clinical practice.

Uncertainty about completeness of endoscopic resection of T1 CRC often creates a treatment dilemma whether or not to operate. In literature, local recurrence rates of only 1% for completion transanal surgery after previous incomplete polypectomy of low-risk T1 rectal cancer have been reported after a median follow-up of 47 months (range 11–109) [42]. Extrapolation of these results to the colorectum would suggests a low rate of local tumor recurrence after local scar excisions following incomplete resection of low-risk T1 CRC. However, in a more recent retrospective study a local recurrence rate of 4.5% was found after additional full-thickness resection of the scar of presumed low risk T1 CRC. Importantly, 75% of these cases turned out to have a high-risk factor after histological review. The abovementioned study has also shown an alarming high percentage of LNM (8.5%) in surgical specimen after previous incomplete resected, presumed low-risk T1 CRC. However, after retrospective histological revision, one or more high-risk factors for LNM were found in 90% of cases with LNM [26]. Since histologic risk assessment in incomplete resected specimen is known to be difficult due to fragmentation and cauterization, certain high-risk factors can potentially be missed. In daily practice, histological double reading in T1 CRC is not routinely performed and assessment of high-risk features is known to have a large interobserver variability between pathologists [43]. Besides, tumor budding is currently not routinely assessed neither reported in pathology reports [27]. To avoid inclusion of misdiagnosed high-risk cases that would require oncological resection our study employs strict in- and exclusion criteria, and all histologic specimens will be centrally revised by an expert pathologist.

Although we believe completion eFTR may reduce surgical overtreatment with substantial health (and economic) impact, the oncological safety of this strategy is yet unclear and requires careful prospective study. At this point, a standard surveillance strategy is lacking. By implementing a strict surveillance protocol, we expect that tumor recurrences can be detected at a still curable stage. This is supported by the results of a recent retrospective study that showed that additional scar excision combined with surveillance and followed by salvage surgery in case of cancer recurrence has a similar 5-year overall survival and metastatic-free survival as compared to direct completion surgery [26]. Given that all patients in this study will be followed for a total duration of five years, we are able to evaluate both short-term (i.e. 2 years) and long-term (i.e. 5 years) oncological safety for completion eFTR.

In conclusion, the current shift towards more detected T1 CRC due to effective CRC screening programs calls for further optimization of treatment for T1 CRC and reduce unnecessary surgery. The LOCAL study is the first study to address feasibility and long-term oncologic safety of additional scar excision with eFTR following a previous incomplete resection of a low-risk T1 CRC in a prospective national cohort study.

## Data Availability

The datasets generated and/or analysed during this study are not publicly available due to individual privacy but are available from the corresponding author on reasonable request.

## Abbreviations

CRC: Colorectal cancer
CT: Computed tomography
eFTR: Endoscopic full-thickness resection
EMR: Endoscopic mucosal resection
ESD: Endoscopic submucosal dissection
FTRD: Full-thickness resection device
IC: Informed consent
LNM: Lymph node metastasis
MRI: Magnetic resonance imaging
R0: Tumor-free lateral and basal resection margins
R1: Positive resection margins
Rx: Indeterminate resection margins

## Declarations

### Trial status

Recruitment of participants is ongoing since January 2021 and aims to be completed July 2024.
